# Granulomatose avec polyangéite du sujet âgé: à propos de deux cas et revue de la literature

**DOI:** 10.11604/pamj.2015.20.341.6618

**Published:** 2015-04-10

**Authors:** Olfa Berriche, Sonia Hammami, Fatma Larbi ammari, Wafa Alaya, Wassia kessomtini, Wafa Chebbi

**Affiliations:** 1 Service de Médecine Interne, Hopital Taher Sfar, Mahdia, Tunisie; 2 Service de Médecine Interne, Hopital Fattouma Bourguiba, Monastir, Tunisie; 3 Unité De Médecine Physique, Hopital Taher Sfar, Mahdia, Tunisie

**Keywords:** Granulomatose, sujets âgés, vascularite, Granulomatosis, elderly, vascularitis

## Abstract

La granulomatose avec polyangéite (GPA) est une vascularite nécrosante des vaisseaux de petit calibre. L’âge moyen d'entrée dans la GPA est entre 35 et 55 ans, les formes gériatriques sont cependant rares, Nous rapportons deux cas de GPA révélés après 60 ans, le mode de révélation était inhabituel, ophtalmologique dans le premier cas et cutané dans le deuxième cas.

## Introduction

La GPA, nouveau terme utilisé pour nommer la classique granulomatose de Wegener, est une vascularite nécrosante des vaisseaux de petit calibre, caractérisée par la fréquence des atteintes ORL et pulmonaires [1]. Elle fait partie des vascularites systémiques touchant les vaisseaux de petit calibre, selon la classification adoptée lors de la conférence de consensus de Chapel Hill [2]. La GPA est caractérisée par une réaction inflammatoire granulomateuse autour de la paroi artérielle et s'accompagne, dans la majorité des cas, de la présence d'autoanticorps circulants, dirigés contre le cytoplasme des polynucléairesneutrophiles (ANCA). Si le diagnostic de la maladie est habituellement fait au cours des quatrièmes et cinquièmes décennies [3], des formes apparues après 60 ans ont déjà été décrites [4]. Nous rapportons deux cas de GPA diagnostiquées après 60 ans.

## Patient et observation

### Cas n°1

**Figure 1:**
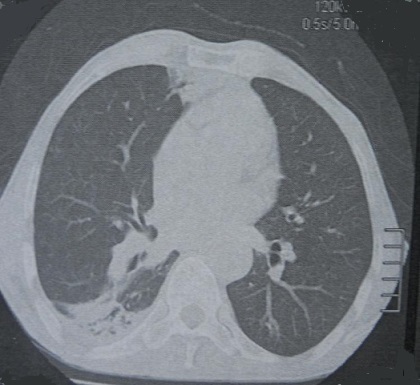
TDM thoracique en coupes axiales: dilatation des bronches, condensation rétractile du segment apical du lobe moyen et de la lingula, et nodule centimétrique angio-centré

Un Patient âgé de 61 ans, sans antécédents pathologiques notables, hospitalisé pour bilan étiologique d'une scléro-uvéite granulomateuse bilatérale, associée à une altération de l’état général. A l'interrogatoire: le patient rapportait la notion de douleur et de rougeur oculaire d'aggravation progressive, le tout évoluant depuis 1 an. L'examen physique à l'admission révélait une température à 37°C, une tension artérielle à 130/80 mm Hg, une pâleur cutanéo-muqueuse L'examen ORL, pleuro-pulmonaire et cardiaque était normal. Le bilan biologique montrait une anémie normochrome normocytaire régénérative (Hb à 11 g/dl, VGM à 93 fl, TCMH à 33,5pg, taux de réticulocytes à 180.000 éléments/mm^3^), un test de Coombs direct négatif, une CRP négative, une vitesse de sédimentation à 56 à la première heure, le bilan rénal montrait une créatinine à 96 mmol/l, une protéinurie de 24 heures négative, et l'ECBU était normal. Le bilan phosphocalcique, l'ionogramme sanguin, le dosage de l'enzyme de conversion de l'angiotensine étaient normaux. La sérologie syphilitique, La recherche de mycobactéries dans les crachats et les urines ainsi que l'intra dermo réaction étaient négatives. Le bilan immunologique objectivait la présence d'ANCA avec une fluorescence cytoplasmique (c-ANCA) et une spécificité anti-PR3 à un titre élevé (> 200 U), suggérant fortement une GPA. La tomodensitométrique thoracique montrait une dilatation des bronches avec une condensation rétractile du segment apical du lobe moyen et de la lingula d'allure infectieuse, et un nodule centimétrique angio-centré (Figure 1). La tomodensitométrie du massif facial a trouvé un épaississement minime du bas fond des sinus maxillaires d'allure non spécifique, une déviation droite de la cloison nasale avec un éperon osseux et une conchabullosa bilatérale (pneumatisation des cornets moyens). La biopsie nasale n'a pas montré de lésions spécifiques. Le diagnostic de GPA était retenu devant la présence de 2 critères de l'ACR: l'atteinte ORL, les anomalies de la radiographie pulmonaire (nodules). Le five factors score 2009 était à 0. Le patient était traité par corticothérapie orale à base de prédnisone à la dose de 1mg/kg/j pendant un mois, avec diminution progressive des doses jusqu’à maintenir la dose de 10 mg/j associé à des bolus bimensuels de cyclophosphamide (Endoxan^®^) à la dose de 600mg/m^2^ à J0, J14, J28 puis 0,7 g/m^2^ tous les 21 jours. Le patient a reçu au total 6 bolus de cyclophosphamide relayés par l'azathioprine (Imurel^®^) à la dose de 2mg/kg/j. Du cotrimoxazole à la dose de 2 cp/j était également prescrit. L’évolution ultérieure était favorable sans rechute avec un recul de 24 mois.

### Cas n°2

Une patiente âgée de 73 ans; aux antécédents de diabète de type2, était hospitalisée pour un purpura vasculaire extensif et une fièvre évoluant depuis 2 semaines. A l'interrogatoire: notion de toux sèche, de rhinorrhée purulente bilatérale et de des poly arthralgies des grosses articulations de type inflammatoire. L'examen physique trouvait une patiente fébrile à 38°, un purpura pétéchial au niveau des membres inférieurs et des bras, des œdèmes blancs mous gardant le godet au niveau des membres inférieurs et des râles crépitants aux deux champs pulmonaires à l'auscultation. L'examen ORL montrait une muqueuse nasale congestive avec des stigmates de saignement. L'auscultation cardiaque était normale et l'examen neurologique était sans particularités. Le bilan biologique montrait une anémie normochrome normocytaire arégénérative (H b à 8,1 g/dl; VGM à 86fl, TCMH à 30pg et un taux de réticulocytes à 70000), un syndrome inflammatoire biologique (VS à 105 à H1, CRP à 36g/l et fibrinogène à 5 g/l), une hypothyroïdie (FT4 à 8,7 p mol/let TSH à12,5mUI/l). Le bilan rénal montrait une créatinine à 353 µmol/l, une hématurie microscopique, la recherche de protéines dans les urines était négative. Les c-ANCA étaient positifs de type anti-PR3. Les hémocultures, le bilan tuberculeux, les sérologies des hépatites B et C, le dosage de la cryoglobulinémie, les AAN et le FR étaient négatifs. L’échographie cardiaque était normale. A la radiographie de thorax elle avait un syndrome alvéolaire bilatéral. La biopsie des fosses nasales objectivait une inflammation crouteuse non spécifique. La biopsie cutanée objectivait une vascularite leucocytoclasique, avec immunofluorescence directe négative. La biopsie rénale mettait en évidence des lésions de vascularite avec suffusions hémorragiques au niveau de la médullaire associée à une réaction granulomateuse sans nécrose. Le diagnostic de GPA était retenu devant la présence de 4critères de l'ACR: l'atteinte ORL, les anomalies à la radiographie pulmonaire, l'hématurie et l'inflammation granulomateuse à la biopsie rénale. Le five factor scores 2009 était à 1. Un traitement par corticothérapie à base de méthylprednisolone à la dose de 15 mg/kg/j a été débuté pendant 3 jours, relayé par prédnisone à la dose de 1mg/kg/j pendant un mois, avec diminution progressive des doses, associée à des bolus bimensuels de cyclophosphamide en intraveineux à la dose de 600 mg/m^2^ à J0, J14 et J28 puis à la dose de 0,7mg/m^2^ tous les 21 jours. Du cotrimoxazole à la dose de 2 cp/j était également prescrit. L’évolution était favorable sous corticothérapie sans rechute avec un recul de 5 ans.

## Discussion

La GPA est une maladie systémique grave d'origine indéterminée caractérisée par son triple tropisme ORL, pulmonaire et rénal. Son diagnostic repose sur des arguments cliniques, biologiques et histologiques. La GPA reste une maladie rare, survenant en général chez des patients âgés de 40 à 50 ans [5] et se voit rarement chez le sujet âgé comme en témoigne la rareté des séries gériatriques publiées [6, 7]. Le diagnostic est souvent porté plus tardivement dans cette tranche d’âge, les différentes séries de la littérature retrouvent en effet un délai diagnostique moyen de cinq à 13 mois après le début des premiers symptômes [4, 6]. Ce délai long était constaté chez notre premier patient, en effet il avait une symptomatologie oculaire trainante depuis un an. Ce délai diagnostique généralement long chez le sujet âgé pourrait être expliquée par l'atypie du tableau initial pouvant rendre le diagnostic plus difficile, l'existence généralement d'une comorbidité associée à cet âge, les formes ORL localisées dont l'expression clinique est fréquemment abâtardie par les antibiotiques et la corticothérapie locale itérative utilisée également dans les formes oculaires. Les signes inauguraux de la GPA chez l'adulte sont multiples et peuvent être à type de manifestations ORL dans 73 à 83% des cas, articulaires dans 20 à 60% des cas et pulmonaires dans 20 à 45% des cas. Les autres manifestations étant plus rares: les manifestations oculaires surviennent dans 7 à 41% des cas et les manifestations cutanées dans 13 à 46% [8], ces deux types de manifestations ont par contre inauguré le tableau clinique chez nos deux patients âgés. Au cours de la GPA, l'atteinte oculaire peut atteindre les différentes structures de l’œil et ses annexes. Elle est rarement inaugurale comme c’était le cas pour notre premier patient et s'associe presque constamment à des lésions des voies aériennes supérieures [9]. Les lésions dermatologiques sont très variables et aspécifiques au cours de la GPA. Elles dominent rarement les signes cliniques et s'intègrent le plus souvent dans le cadre d'une atteinte multiviscérale [10], comme le cas de notre patiente âgée de 73 ans qui avait simultanément une atteinte ORL, rénale et pulmonaire. Par ordre de fréquence décroissante, on peut observer au cours de la GPA, des lésions de purpura infiltré et nécrotique, des ulcérations de la muqueuse buccale, des nodules sous-cutanés siégeant aux coudes et des ulcérations cutanées [11]. Pour Vassalo et al [12], Les manifestations ophtalmologiques et cutanées de la maladie diminuaient en fréquence avec l’âge, ceci n'a pas été le cas pour nos deux patients âgés de plus de 60 ans et qui avaient dès le début et respectivement ces deux types d'atteinte.

Le dépistage de l'atteinte rénale revêt un intérêt majeur au cours de la GPA étant donné son impact pronostique sur la survie des patients. La prévalence de la néphropathie dans la GPA est variable, allant de 45 à 90% des cas selon les séries, variant selon l'origine du recrutement des patients, la définition retenue pour étiqueter la maladie rénale et la durée du suivi. Effectivement dans la série de Hoffman [5], seuls 18% des patients avaient des signes néphrologiques lors du diagnostic initial de la GPA, mais après un suivi de quelques années, près de 80% d'entre eux développaient l'atteinte rénale. Différents travaux font état d'une prévalence accrue de l'atteinte rénale de la GPA chez les sujets âgés [4, 6, 13], l'atteinte rénale était en effet présente chez notre deuxième patiente et découverte lors d'un bilan rénal de dépistage dans le cadre de sa maladie, elle était absente dans le premier cas. Dans une série gériatrique publiée par Fauchais et al [7] incluant 11 patients âgés de plus de 60 ans et suivis pour GPA, l'atteinte ORL était observée dans 91% des cas et l'atteinte pulmonaire était aussi fréquente touchant 82% des cas. Les atteintes ORL et pulmonaire étaient en effet constantes dans nos deux observations. L’âge avancé constitue aussi un facteur pronostique de poids au cours de la GPA, il constitue un élément important à prendre en compte, notamment lors du choix du traitement immunosuppresseur, puisque plusieurs études semblent évoquer que la forte mortalité des patients de plus de 65 ans était en grande partie secondaire aux effets secondaires des immunosuppresseurs [14, 15], pour le cas de nos deux patients l’évolution après traitement corticoïde et immunosuppresseur était favorable. Si un traitement immunosuppresseur précoce et intense s'impose compte tenu de la gravité potentielle de la maladie, la défervescence thérapeutique pourrait être plus précoce dans ce groupe de patients compte tenu de la fréquence significativement plus élevée d’épisodes infectieux sévères lors du traitement d'entretien.

## Conclusion

La granulomatose avec polyangéite, est une maladie systémique sévère. Son diagnostic, pas toujours facile pour le clinicien non sensibilisé à ce type de maladies, repose sur des signes cliniques non spécifiques mais souvent assez bruyants (notamment au niveau ORL, pulmonaire), sur des anomalies histologiques caractéristiques et la présence quasi constante d'un auto-anticorps spécifique, les c-ANCA anti-PR3. Nos deux observations gériatriques illustrent une présentation initiale atypique de la GPA rendant le diagnostic plus difficile, et mettent le point sur la nécessité d’établir un bilan lésionnel multi viscéral dès la suspicion diagnostique de la maladie car le pronostic vital peut être mis en jeu.
